# Progesterone resistance in atypical endometrial hyperplasia: Expression and mechanisms of hormone-responsive molecules

**DOI:** 10.1097/MD.0000000000048452

**Published:** 2026-05-01

**Authors:** Zhixiang Huang, Tingzhou Jiang, Jun Yao, Zhengping Tian, Zhuo Liang, Zhong Lin, Pinxiu Huang

**Affiliations:** a Clinical Medical College, Guilin Medical University, Guilin, China; b Center of Reproductive Medicine, The First Affiliated Hospital of Guilin Medical University, Guilin, China; c Physical Examination Center, Laibin People’s Hospital, Laibin, Guangxi, China; d Center of Reproductive Medicine, Guangxi Zhuang Autonomous Region Reproductive Hospital, Nanning, China.

**Keywords:** atypical endometrial hyperplasia, estrogen receptor alpha, progesterone receptor, progesterone resistance, SOX7

## Abstract

This study aimed to delineate the molecular profile underlying progesterone resistance in atypical endometrial hyperplasia (AEH), a precancerous condition associated with a high risk of malignant transformation. Using a retrospective cohort of 20 AEH patients who completed a 6-month progestin therapy, we compared protein expression between 10 progesterone-resistant and 10 progesterone-sensitive tissues using immunohistochemistry and Western blot analysis. The results revealed a distinct molecular signature in resistant tissues, characterized by significant upregulation of estrogen signaling components (ERa, pS2, and MUC1) and proliferation markers (SOX7 and Ki-67). Concurrently, the key progesterone receptor signaling elements (PR, FKBP4, FKBP5, and FOSL2) were markedly downregulated. These findings indicate that progesterone resistance is associated with sustained activation of estrogen-driven proliferative pathways coupled with impaired progesterone signaling, leading to unabated cellular growth. The coordinated dysregulation of these hormone-responsive and proliferation-related molecules highlights a fundamental hormonal imbalance and proliferative disruption in progesterone-resistant AEH. This study provides a molecular framework for understanding progesterone resistance and suggests potential targets, such as SOX7, for future therapeutic strategies aimed at restoring hormonal sensitivity and controlling disease progression in conservative fertility-sparing management.

## 1. Introduction

Atypical endometrial hyperplasia (AEH) is a precancerous condition characterized by abnormal endometrial thickness, excessive proliferation of the endometrial glands, and varying degrees of cellular atypia.^[1]^ It commonly presents with abnormal uterine bleeding (AUB), including menstrual irregularities and prolonged or heavy periods.^[2]^ In addition, some patients may experience dull abdominal pain or a sensation of weight in the lower abdomen. Endometrial hyperplasia can be classified into 3 categories: simple, complex, and atypical. AEH is the most severe form and is distinguished by both cellular atypia and abnormal glandular architecture. As AEH has a high risk of progression to endometrial cancer,^[3]^ timely diagnosis and management are essential.

The endometrium is a dynamic, hormone-sensitive tissue that undergoes cyclical proliferation and differentiation under the precise regulation of estrogen and progesterone. Estrogen promotes endometrial cell proliferation, whereas progesterone antagonizes estrogenic effects by inducing secretory transformation and suppressing estrogen receptor expression.^[4]^ Disruption of this hormonal equilibrium – commonly resulting from unopposed estrogen stimulation or impaired progesterone action – is a fundamental driver of endometrial hyperplasia.^[4]^ In AEH, such hormonal imbalance is particularly pronounced, fostering a pro-proliferative milieu that facilitates malignant transformation.

Endometrial cancer is one of the most common gynecological cancers, and its incidence is increasing. Conservative treatment for endometrial cancer and AEH is essential to preserve fertility.^[2]^ Progestin therapy is a widely used treatment for atypical endometrial hyperplasia and early stage endometrial cancer, especially in women who want to maintain fertility.^[5]^ Progestins work by binding to progesterone receptors (PR), slowing disease progression, and inhibiting estrogen-induced excessive endometrial growth. Progestogen resistance is defined as the failure of progestin therapy to effectively control the disease after 6 months of treatment.^[6]^ The inability of progesterone to successfully counteract the proliferative effects of estrogen, resulting in endometrial hyperplasia that persists or worsens, is known as progesterone resistance. This increases the risk of endometrial cancer and reduces treatment effectiveness. Reduced progesterone receptor transcriptional activity, increased estrogen receptor activation, and aberrant expression of cellular proliferation factors such as SOX7 are the main processes that generate progesterone resistance^.[7]^ Progesterone resistance is frequently observed in endometriosis (EMS), polycystic ovarian syndrome (PCOS), and other gynecological diseases when treating endometrial hyperplasia.

The precise molecular processes of progesterone resistance are still not fully understood, despite the large number of studies investigating the therapeutic responses and clinical manifestations linked to the condition.^[8]^ According to current studies, progesterone resistance may be significantly influenced by ERa, the central receptor in the estrogen signaling cascade. By controlling downstream molecules, including pS2 and MUC1, ERa contributes to cellular proliferation and may have aberrant interactions with the progesterone signaling pathway.^[9]^ Progesterone resistance may also be significantly influenced by PR dysfunction, namely the downregulation of its chaperone proteins FKBP4 and FKBP5, as well as the transcription factor FOSL2.^[10]^ Additionally, it has been shown that SOX7, a transcription factor linked to cell proliferation, controls the Wnt/β-catenin signaling pathway and may encourage proliferation during progesterone resistance^.[11]^

By comparing molecular differences between the progesterone-resistant group and the control group after 6 months of progesterone treatment, this study sought to thoroughly investigate the expression variations of important molecules, such as ERa, PR, pS2, MUC1, FKBP4, FKBP5, FOSL2, and SOX7^.[12]^ This study aimed to investigate the molecular processes underlying progesterone resistance and to offer a theoretical framework for therapeutic intervention. This work intends to offer new insights for the diagnosis and treatment of progesterone resistance through a thorough understanding of these molecular pathways.^[13]^

## 2. Materials and methods

### 2.1. Study design and patient cohort

This was a retrospective cohort study conducted at a single center. The study period spanned from January 2019 to December 2022. The study was approved by the Ethics Committee of Liuzhou Maternal and Child Health Hospital (Approval No.: Fast Review-Research-2021-079) and conducted in accordance with the Declaration of Helsinki. Written informed consent was obtained from all the participants.

Patient screening and enrollment were summarized as follows: 108 patients were initially screened. The exclusion criteria applied prior to treatment were as follows: patients diagnosed with AEH but not treated with progestin therapy (n = 25); patients who received other pharmacological or biological treatments in addition to progestin therapy (n = 31); and patients with other systemic diseases and/or concurrent gynecology-related cancers (n = 18). Consequently, 34 eligible patients were enrolled and received oral medroxyprogesterone acetate (MPA) therapy at a dose of 160 mg/day for 6 months.

Posttreatment grouping was performed as follows: after the 6-month MPA treatment, patients were excluded due to loss to follow-up (n = 5), disease progression, or the need for combination therapy (n = 8). The remaining 21 patients were categorized based on the pathological examination results into a progesterone-sensitive group (n = 10) and a progesterone-resistant group (n = 11). One patient in the resistant group was later excluded because of specimen handling oversight, resulting in a final cohort of 10 patients in each group for immunohistochemical (IHC) analysis. Because tissue samples were kept for 3 years before Western blot (WB) analysis, 4 samples from each group were excluded because of protein degradation during storage, leaving 6 samples from each group for the final analysis. Owing to the retrospective nature of the study and the rarity of well-documented progesterone-resistant cases, a convenience sample of all eligible patients during the study period was used. Importantly, at present, there are no reports on the probability of progesterone resistance in atypical hyperplasia of the endometrium, and there are more than 15 cases between January 2019 and December 2022.

The baseline demographic and clinical characteristics of the 2 groups were comparable (Table S4, Supplemental Digital Content, https://links.lww.com/MD/R757).

### 2.2. Variable definition

The primary outcome was progesterone resistance, defined as the persistence of hyperplastic lesions after 6 months of medroxyprogesterone acetate therapy. This 6-month cutoff was chosen based on prior clinical studies and guidelines, although we acknowledge the absence of universally standardized response criteria. The key exposure variables were the protein expression levels of estrogen signaling molecules (ERα, pS2, and MUC1), progesterone signaling molecules (PR, FKBP4, FKBP5, and FOSL2), and proliferation markers (SOX7 and Ki-67). The primary antibodies and their dilution concentrations are shown in Table S1, Supplemental Digital Content, https://links.lww.com/MD/R757. The reagents and consumables are shown in Table S2, Supplemental Digital Content, https://links.lww.com/MD/R757. The major Instruments and Equipment are shown in Table S3, Supplemental Digital Content, https://links.lww.com/MD/R757.

### 2.3. Immunohistochemistry (IHC) and quantification

#### 2.3.1. Tissue preparation and sectioning

Endometrial tissues were rinsed in PBS, fixed in 4% paraformaldehyde, embedded, and sectioned at 4 to 6 μm. The sections were flattened in warm water (40–50°C), mounted, and dried at 60°C for 30 minutes.

#### 2.3.2. Deparaffinization, rehydration, and antigen retrieval

Sections were deparaffinized in xylene (2 × 5 min), rehydrated with graded ethanol, and rinsed in tap water (2 × 5 min). Antigen retrieval was performed by microwave heating in citrate buffer (pH 6.0) (boiled, then sub-boiled for 10 minutes). After cooling, sections were washed with PBS (2 × 5 min).

#### 2.3.3. Immunostaining

Endogenous peroxidase was blocked with 3% H_2_O_2_ (dark, 10 minutes) followed by washing with tap water (2 × 5 min). Sections were incubated with 5% normal goat serum (RT, 1 hour) to block non binding, followed by incubation with primary antibodies (4°C overnight; see Table S1, Supplemental Digital Content, https://links.lww.com/MD/R757). After washing with PBS (2 × 5 min), the sections were incubated with HRP-conjugated secondary antibodies (RT, 1 hour), washed again (PBS, 2 × 5 min), and visualized using a DAB substrate kit. The reaction was stopped by adding tap water.

#### 2.3.4. Counterstaining and mounting

Sections were counterstained with hematoxylin (30 seconds), rinsed with tap water, dehydrated in graded ethanol, cleared in xylene, and coverslipped with a neutral resinous mounting medium.

#### 2.3.5. Image acquisition

Stained sections were examined under a light microscope, and representative images were captured for analysis.

### 2.4. Western blot analysis

#### 2.4.1. Protein extraction and quantification

Frozen tissues were washed (ice-cold PBS, 3×), homogenized in chilled RIPA buffer containing protease inhibitors, incubated on ice (30 minutes), and centrifuged (12,000 × g for 20 minutes at 4°C). The supernatants were collected and stored at − 80°C. Protein concentration was measured by BCA assay: samples/standards were mixed with BCA reagent in a 96-well plate, incubated (37°C for 30 minutes), and read at 562 nm. The samples were then mixed with 5 × SDS loading buffer (4:1), boiled (95–100°C, 10 minutes), and stored at − 80°C.

#### 2.4.2. Electrophoresis and transfer

Equal protein amounts (20–40μg/lane) were separated by SDS-PAGE (10% separating, 5% stacking gel) at 80 V (until the dye front entered the separating gel) and 120 V. Proteins were wet-transferred to methanol-activated PVDF membranes (13 V, 45 minutes).

#### 2.4.3. Immunoblotting

Membranes were blocked (5% nonfat milk in PBST, 2 hours, RT), incubated overnight at 4°C with primary antibodies (Table S1, Supplemental Digital Content, https://links.lww.com/MD/R757; GAPDH as loading control), washed (PBST, 5 × 5 min), incubated with HRP-conjugated secondary antibodies (1:5000, 2 hours, RT), washed again, and visualized using ECL. Signals were captured using a ChemiDoc system.

### 2.5. Statistical analysis

Statistical Analysis. Three nonfields (200 × magnification) from the central lesional area of each section were selected to avoid edges, necrotic areas, and large vessels. Mean optical density was measured (ImageJ v1.53) and averaged for each sample. Two independent investigators performed acquisition and measurements (not blinded). Data are mean ± SD. Normality and homogeneity of variance were confirmed (*P* > .05). Group comparisons were performed using unpaired two-tailed t-tests (SPSS v29.0). Statistical significance was set at *P* < .05. As this exploratory study evaluated 9 markers, no multiple comparison correction was applied. Missing data were not imputed, and only complete data were analyzed.

**Informed Consent** Written informed consent was obtained from all the participants prior to their inclusion in the study.

## 3. Results

### 3.1. IHC reveals imbalanced hormone signaling and enhanced proliferation in progesterone resistance

**Figure 1.**
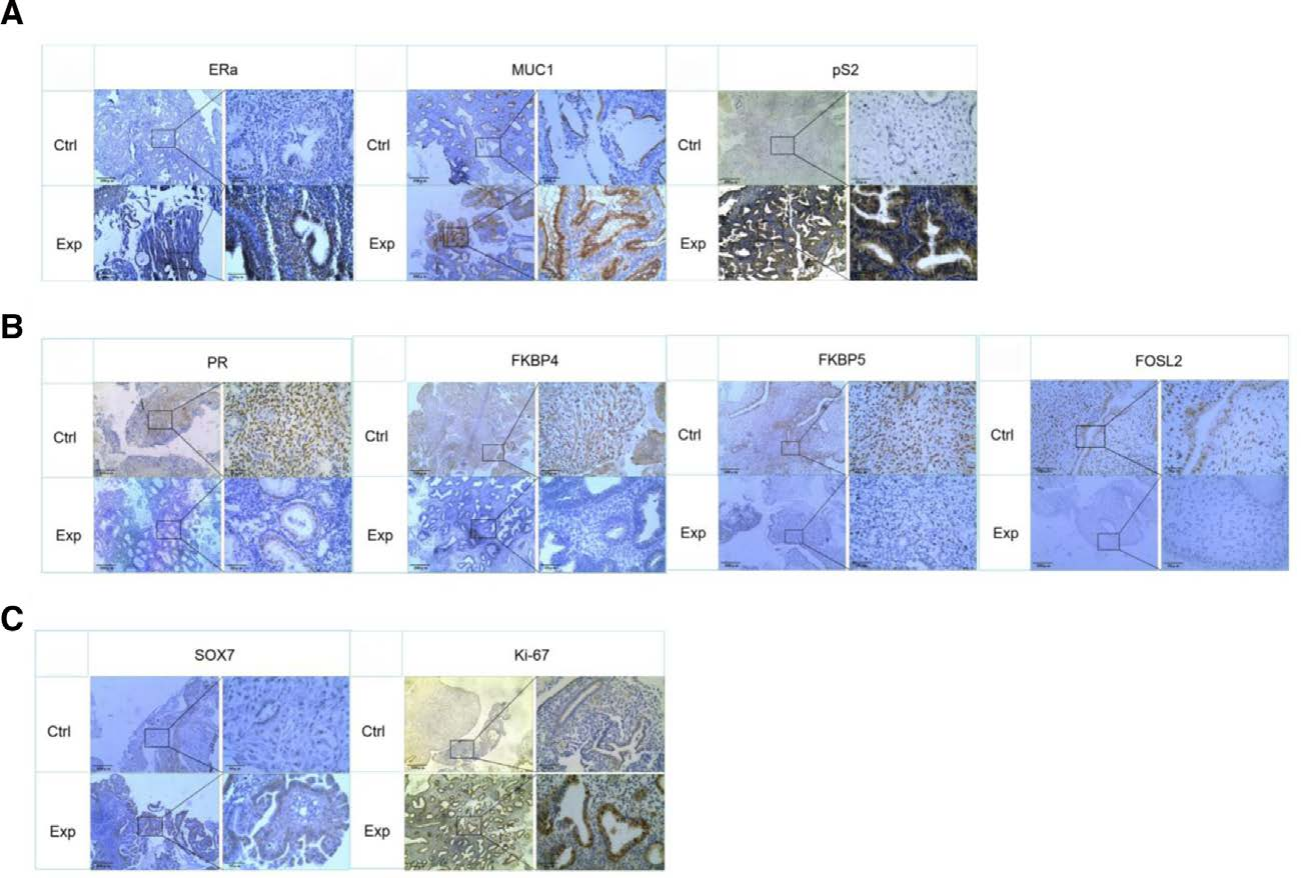
Immunohistochemical analysis reveals coordinated dysregulation of estrogen signaling, progesterone signaling, and proliferation pathways in progesterone-resistant AEH.

Immunohistochemical analysis revealed distinct molecular profiles that distinguished progesterone-resistant from progesterone-sensitive tissues, and the components of estrogen signaling, specifically nuclear ERα, cytoplasmic pS2, and membrane-associated MUC1, were significantly elevated in PR-resistant specimens (Fig. 1A). These upregulation patterns were strongly supported by quantitative analysis, highlighting the widespread activation of estrogen-responsive pathways in the resistant phenotype (Table 1).

**Table 1:** Expression and statistical analysis of ERα, MUC1, pS2and PR、FKBP4、FKBP5、FOSL2 and SOX7、Ki-67 in control and experimental groups.

Parameters	Group	n	MOD Value (Mean ± SD)	t	*P*
ERɑ	Ctrl	10	0.31123 ± 0.00166	24.97	*P* < .001
	Exp	10	0.49926 ± 0.00201		
MUC1	Ctrl	10	0.18640 ± 0.000994	19.41	*P* < .001
	Exp	10	0.35320 ± 0.00596		
pS2	Ctrl	10	0.21674 ± 0.001187	39.47	*P* < .001
	Exp	10	0.72460 ± 0.009672		
PR	Ctrl	10	0.36469 ± 0.002323	13.41	*P* < .001
	Exp	10	0.23048 ± 0.001885		
FKBP4	Ctrl	10	0.35477 ± 0.00063	31.63	*P* < .001
	Exp	10	0.18711 ± 0.001031		
FKBP5	Ctrl	10	0.34629 ± 0.00241	14.40	*P* < .001
	Exp	10	0.20389 ± 0.000672		
FOSL2	Ctrl	10	0.30551 ± 0.00255	34.90	*P* < .001
	Exp	10	0.18952 ± 0.00193		
SOX7	Ctrl	10	0.20830 ± 0.000566	24.18	*P* < .001
	Exp	10	0.32283 ± 0.001503		
Ki-67	Ctrl	10	0.27917 ± 0.00211	13.24	*P* < .001
	Exp	10	0.55725 ± 0.003982		

ERα, MUC1, pS2and PR、FKBP4、FKBP5、FOSL2 and SOX7、Ki-67 all with *P* < .05, showing statistically significant differences.

*(A) Expression profiles of estrogen signaling molecules: ERa, MUC1, and pS2.are significantly upregulated in progesterone-resistant tissues. (B) Expression patterns of progesterone signaling components: PR, FKBP4, FKBP5, and FOSL2.are coordinately downregulated. (C) Localization of proliferation-associated markers: SOX7 and Ki-67.show markedly elevated expression in resistant tissues. *Left panels: 40 × magnification (scale bar: 200 μm); right panels: 200 × magnification (scale bar: 50 μm).

In contrast, PR resistant tissues showed significantly reduced progesterone signaling. Nuclear PR expression was found to decrease with cell type, particularly in the stromal compartment. Coordinated downregulation of the crucial co-chaperones FKBP4 (nuclear/cytoplasmic) and FKBP5 (nuclear), as well as the downstream transcriptional regulator FOSL2 (nuclear), coincided with this receptor-level reduction (Fig. 1B). These significant decreases were confirmed by statistical analysis, which is in line with the thorough disruption of the progesterone signaling axis (Table 1).

Further divergences emerged in the assessment of proliferative drive. The transcription factor SOX7 and canonical proliferation marker Ki-67 both demonstrated significantly intensified nuclear expression in resistant tissues. This pattern denotes a persistently hyperproliferative cellular state unabated by progestin intervention (Fig. 1C). Quantification validated this sustained proliferative effect, highlighting the failure of growth suppression in the resistant group (Table 1).

### 3.2. Western blot analysis confirms the protein expression trends

**Figure 2.**
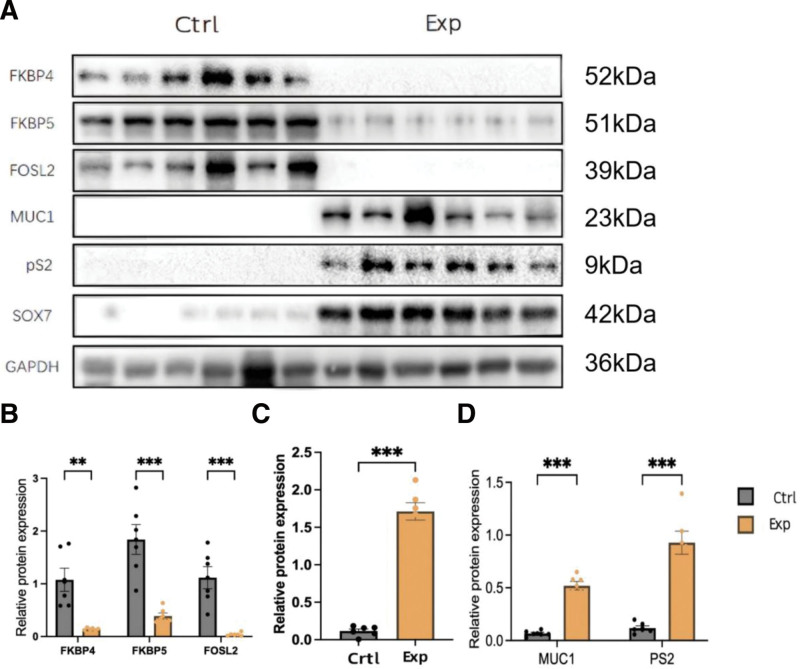
Western blot analysis validates the protein expression trends in progesterone-resistant AEH tissues.

Western blot analysis was performed to confirm the expression patterns identified by IHC (Fig. 2A). According to densitometric measurements, the progesterone-responsive factors FKBP4, FKBP5, and FOSL2 were consistently downregulated in progesterone-resistant tissues. Concurrently, the proliferation-associated protein SOX7 and estrogen-responsive effectors (pS2 and MUC1) were significantly upregulated (Figs. 2B–2D). Every change was statistically significant (*P* < .05), supporting the progesterone-resistant state’s intrinsic protein level dysregulation.

* (A) Representative Western blot images showing protein expression levels of the indicated molecules in progesterone-sensitive (S) and progesterone-resistant (R) groups. GAPDH/β-actin was used as a loading control. (B) Densitometric quantification of progesterone receptor-responsive molecules (FKBP4, FKBP5, FOSL2) confirms their significant downregulation in the resistant group. (C) Densitometric quantification of SOX7 protein expression confirms its significant upregulation in the resistant group. (D) Densitometric quantification of estrogen receptor-responsive molecules (pS2, MUC1) confirms their significant upregulation in the resistant group. *Data are presented as mean ± SD (n = 6). *P < .05, **P < .01, **P < .001.

## 4. Discussion

This study provides a comprehensive molecular profile of progesterone-resistant AEH by comparing protein expression patterns between resistant and sensitive tissues after 6 months of progestin therapy. Using immunohistochemistry and Western blot analysis, we identified a coordinated molecular signature characterized by the upregulation of estrogen signaling components (ERα, pS2, MUC1) and proliferation markers (SOX7, Ki-67), along with the downregulation of progesterone signaling elements (PR, FKBP4, FKBP5, FOSL2). While these findings contribute to the growing understanding of progesterone resistance, several important caveats and alternative interpretations must be carefully considered.

### 4.1. Upregulated estrogen signaling: A consistent feature of resistance?

Consistent with prior studies in endometriosis and endometrial cancer, we observed significant upregulation of ERα and its downstream effectors, pS2 and MUC1, in progesterone-resistant AEH tissues.^[14]^ ERα is a master transcriptional regulator of estrogen-driven endometrial proliferation, and pS2 and MUC1 are well-characterized ERα target genes involved in cell survival and epithelial homeostasis.^[15]^ Our findings align with the association between sustained estrogen pathway activation and a poor progestin response. However, it remains unclear whether ERα upregulation is a cause or consequence of resistance. Some studies have reported conflicting findings; for example, certain endometrial cancer cohorts show preserved or even reduced ERα expression in aggressive phenotypes, suggesting context-dependent roles.^[14]^ Thus, while our data support an association between ERα signaling and progesterone resistance, the precise functional contribution warrants further investigation.

Notably, we did not assess estrogen receptor beta (ERβ) or G protein-coupled estrogen receptor (GPER), both of which have been implicated in modulating endometrial hormone sensitivity and may exhibit distinct or opposing roles to ERα.^[16]^ Future studies should examine the full repertoire of estrogen-signaling components.

### 4.2. Impaired progesterone signaling: beyond receptor loss

The coordinated downregulation of PR, FKBP4, FKBP5, and FOSL2 in resistant tissues suggests global impairment of the progesterone signaling axis.^[13]^ PR is the primary mediator of progestin action, and its reduction, particularly in the stromal compartment, is a well-recognized feature of progesterone-resistant endometria.^[10]^ FKBP4 and FKBP5 are immunophilins that function as PR chaperones, facilitating proper receptor folding, nuclear translocation, and transcriptional activity^[17]^; both are PR target genes.^[11,12]^ FOSL2, a component of the AP-1 transcription factor complex, has been implicated in PR-dependent gene regulation and decidualization.^[18]^ We found that FOSL2 is also a PR target gene during human endometrial decidualization. These results indicated decreased PR transcriptional activity in progesterone-resistant AEH tissues. However, several important qualifications are required. First, our study measured only total PR protein expression and did not distinguish between the PR-A and PR-B isoforms, which have distinct transcriptional activities and may be differentially regulated in resistance.^[19]^ These mechanistic hypotheses remain speculative and require direct experimental testing using in vitro models with PR-responsive reporter assays, chromatin immunoprecipitation, and protein-protein interaction studies.^[20]^

### 4.3. SOX7 overexpression: driver or bystander in progesterone resistance?

A notable finding of this study was the significant upregulation of SOX7 in progesterone-resistant tissues, concomitant with elevated Ki-67 expression. SOX7 is a transcription factor that has been reported to both promote and suppress proliferation, depending on the cellular context.^[21]^ In colorectal and lung cancers, SOX7 acts as a tumor suppressor by inhibiting Wnt/β-catenin signaling. Conversely, in the endometrial context, emerging evidence suggests that SOX7 may promote proliferation and oppose progesterone-driven differentiation.^[22]^ This apparent discrepancy highlights the importance of tissue- and disease-specific functional studies.

Our data demonstrate a statistical association between SOX7 expression and progesterone, but provide no direct evidence that SOX7 drives proliferation or impairs PR signaling in AEH. The hypothesis that SOX7 contributes to resistance via Wnt/β-catenin activation or cross talk with PR signaling pathways is biologically plausible but currently conjectural. Functional studies, including SOX7 knockdown or overexpression in endometrial epithelial and stromal cells, combined with the assessment of proliferation, progesterone response, and PR target gene expression, are urgently needed to establish causality.

### 4.4. Placing our findings in context: alternative mechanisms and literature heterogeneity

Progesterone resistance is a multifactorial phenomenon, and our focus on the estrogen, progesterone, and SOX7 pathways represents only a subset of the potential mechanisms. Other proposed contributors include: altered progesterone metabolism via dysregulation of 17β-hydroxysteroid dehydrogenases and AKR1C family enzymes^[23]^; PR isoform switching, with an increased PR-A/PR-B ratio favoring anti-inflammatory and anti-proliferative transcriptional programs^[24]^; microRNA-mediated post repression of PR and its co-regulators^[8]^; stromal-epithelial decoupling, wherein stromal PR loss renders epithelium insensitive to paracrine progestin signals^[25]^; and genetic and epigenetic alterations, including PR promoter methylation and somatic mutations in hormone signaling pathways.^[26]^

Importantly, the published findings on these mechanisms are heterogeneous. While some studies have reported PR downregulation as a consistent feature of progesterone resistance,^[27]^ others have found preserved PR expression but impaired downstream signaling.^[28]^ Similarly, ERα overexpression has not been uniformly observed across resistant cohorts. These discrepancies may reflect differences in disease stage, prior treatment exposure, hormonal milieu, or technical variables, such as antibody specificity and scoring systems. Our findings should be interpreted within this broader and sometimes contradictory evidence landscape.

### 4.5. Study limitations

Several important limitations must be acknowledged beyond those discussed above. First, the small sample size and single-center retrospective design limit generalizability and statistical power, and may have introduced selection bias, particularly through the exclusion of patients with disease progression.^[29]^ It is important to note that progesterone-resistant cases are clinically rare; only 10 eligible resistant patients were identified at our center over a 3 years. Thus, despite its small sample size, this study provides valuable exploratory data on this uncommon but clinically significant condition.

Second, the absence of pretreatment biopsies precludes the assessment of whether the observed molecular profiles pretherapy or emerge during treatment. Third, IHC quantification was not performed blinded to the group assignment, and the selection of 3 fields per section, while standardized, may not fully capture intratumoral heterogeneity. Furthermore, whole-slide digital imaging was not available at our institution at the time of the study, limiting our ability to perform a fully automated quantitative analysis. Fourth, as an exploratory study examining 9 molecular markers simultaneously, we did not adjust for multiple comparisons, and the findings require independent validation in confirmatory cohorts with prespecified hypotheses and appropriate correction. Fifth, no functional experiments were performed and all mechanistic interpretations remained correlational and speculative.

### 4.6. Implications and future directions

Notwithstanding these limitations, this study provides a comprehensive descriptive framework of the molecular alterations associated with progesterone resistance in AEH. The consistent patterns of estrogen pathway upregulation, progesterone pathway downregulation, and SOX7 overexpression identified these molecules as candidate biomarkers for resistance and potential therapeutic targets worthy of further investigation. We propose the following priorities for future research: (i) prospective longitudinal cohort studies with pre- and posttreatment biopsies to distinguish predictive biomarkers from treatment-induced changes; (ii) functional validation of ERα, PR, FKBP4/FKBP5, FOSL2, and SOX7 using in vitro and in vivo models of endometrial hormone response; (iii) mechanistic dissection of putative crosstalk between SOX7 and PR signaling pathways; (iv) evaluation of combination strategies targeting ERα (e.g., selective estrogen receptor degraders) alongside progestins in preclinical models; and (v) multi-center collaborative efforts to assemble adequately powered cohorts for validation and confounding-adjusted analyses.

## 4. Conclusions

In summary, this study demonstrated that progesterone-resistant AEH is characterized by a distinct molecular signature involving coordinated upregulation of estrogen-responsive proteins and proliferation markers, along with downregulation of progesterone signaling components. These findings provide a descriptive molecular foundation for understanding progesterone resistance and generating specific testable hypotheses regarding the potential roles of ERα, PR chaperones, and SOX7 in this process. However, causal relationships, mechanistic pathways, and therapeutic implications need to be established through rigorous functional and translational studies. The results should be interpreted with appropriate caution and viewed as hypothesis generating rather than hypothesis confirming.

## Author contributions

**Writing—original draft:** Zhixiang Huang.

**Methodology:** Tingzhou Jiang.

**Data curation:** Jun Yao.

**Resources:** Zhengping Tian.

**Software:** Zhuo Liang.

**Funding acquisition:** Zhong Lin, Pinxiu Huang.

**Supervision:** Pinxiu Huang.

## Supplementary Material
