# Supplementary material for: Progesterone resistance in atypical endometrial hyperplasia: Expression and mechanisms of hormone-responsive molecules
Source: Medicine (Baltimore). 2026 May 1;105(18):e48452. doi: 10.1097/MD.0000000000048452 (PMC13138396; doi:10.1097/MD.0000000000048452)
Supplement: Supplementary file 1 [file medi-105-e48452-s001.pdf]

Supplementary Table S1: Primary Antibodies and Dilution Concentrations

Antibody	Manufacturer	Dilution Concentration	Catalog Number
ERα	Wuhan Sanchong Biotechnology	1:1000	Cat No. 21244-1-AP
MUC1	Wuhan Sanchong Biotechnology	1:1000	Cat No. 83311-4-RR
pS2	Wuhan Sanchong Biotechnology	1:1000	Cat No. 13734-1-AP
PR	CST	1:1000	#8757
FKBP4	Wuhan Sanchong Biotechnology	1:1000	Cat No. 10655-1-AP
FKBP5	Wuhan Sanchong Biotechnology	1:1000	Cat No. 86189-2-RR
FOSL2	Wuhan Sanchong Biotechnology	1:1000	Cat No. 15832-1-AP
SOX7	Wuhan Sanchong Biotechnology	1:1000	Cat No. 23925-1-AP
Ki-67	Wuhan Sanchong Biotechnology	1:1000	Cat No. 27309-1-AP
GAPDH	Wuhan Sanchong Biotechnology	1:1000	Cat No. 10494-1-AP

Supplementary Table S2: Reagents and Consumables

Reagent/Consumable Name	Brand
IHC Antibody Diluent	Thermo Fisher Scientific Inc
Citrate Buffered Solution (pH 6.0)	Wuxi Aorui Dongyuan Biotechnology Co., Ltd.
Phosphate Buffered Saline Powder (1× PBS, Calcium/Magnesium-Free)	Beijing Regene Biotech Co., Ltd.
Xylene	Sinopec
Anhydrous Ethanol	Shanghai Aladdin Bio-Chem Technology Co., Ltd.
75% Ethanol	Shanghai Aladdin Bio-Chem Technology Co., Ltd.
DAB Chromogen Kit	GeneTech (Shanghai) Co., Ltd.
Hematoxylin Stain Solution	Wuxi Aorui Dongyuan Biotechnology Co., Ltd.
Neutral Gum	Beijing Solabio Technology Co., Ltd.
PAGE Gel Rapid Preparation Kit	Shanghai Yamei Biopharmaceutical Technology Co., Ltd.
RIPA Total Protein Lysis Buffer	Thermo Fisher Scientific Inc.
BCA Protein Assay Kit	Thermo Fisher Scientific Inc.
TBST	Shanghai Yamei Biopharmaceutical Technology Co., Ltd.
Electrophoresis Buffer	Shanghai Yamei Biopharmaceutical Technology Co., Ltd.
Blotting Buffer	Shanghai Yamei Biopharmaceutical Technology Co., Ltd.
Antibody Wash Buffer	Thermo Fisher Scientific Inc.
Developing and Fixing Solution	Thermo Fisher Scientific Inc.
Protein Marker	Thermo Fisher Scientific Inc.
0.45μm PVDF membrane	Millipore
Rapid detachment buffer	Shanghai Yamei Biotechnology Co., Ltd.
Goat serum	Thermo Fisher Scientific Inc.

Supplementary Table S3: Major Instruments and Equipment

Instrument Name	Brand
Microtome	Germany LEICA
Tissue Fixation and Drying Unit	Prisstar (Changzhou) Medical Equipment Co., Ltd.
Microwave Oven	China Supor
Biological Microscope	Germany Leica Microsystems GmbH
Electrophoresis Unit	Bollinger Company, USA
Electrophoresis Tank	Bollinger Company, USA
High-Speed Refrigerated Centrifuge	Eppendorf
Ice Maker	Zhengzhou Haobo Machinery Equipment Co., Ltd.
Horizontal Shaker	Haimen Qilinbel Instrument Manufacturing Co., Ltd.
Dry Bath	Ningbo Yinzhou Qun'an Laboratory Instrument Co., Ltd.
Gel Imaging System	Bollinger Company, USA
Vortex Mixer	Jiangsu Kangjian Medical Supplies Co., Ltd.

Supplementary Table S4: Baseline demographic and clinical characteristics of progesterone-sensitive and progesterone-resistant groups

variable	Non-progesterone resistance group(n=10)	Progesterone resistance group(n=10)	P value
Age	35.51±5.52	36.45±4.67	0.686
BMI	22.52±2.31	22.67±2.56	0.892
Number of pregnancies	2.21±1.05	2.618±1.56	0.502
Number of births	1.05±1.25	1.24±1.50	0.762
